# Use of [^18^F]FDOPA-PET for *in vivo *evaluation of dopaminergic dysfunction in unilaterally 6-OHDA-lesioned rats

**DOI:** 10.1186/2191-219X-1-25

**Published:** 2011-11-10

**Authors:** Kiyoshi Kyono, Tadayuki Takashima, Yumiko Katayama, Toshiyuki Kawasaki, Riyo Zochi, Maki Gouda, Yasuhiro Kuwahara, Kazuhiro Takahashi, Yasuhiro Wada, Hirotaka Onoe, Yasuyoshi Watanabe

**Affiliations:** 1 Advanced Medical Research Laboratories, Mitsubishi Tanabe Pharma Corporation, Osaka, Japan; 2 RIKEN Center for Molecular Imaging Science, Hyogo, Japan

**Keywords:** Parkinson's disease, positron emission tomography, [^18^F]FDOPA, 6-OHDA, dopamine

## Abstract

**Background:**

We evaluated the utility of L-3,4-dihydroxy-6-[^18^F]fluoro-phenylalanine ([^18^F]FDOPA) positron emission tomography (PET) as a method for assessing the severity of dopaminergic dysfunction in unilaterally 6-hydroxydopamine (6-OHDA)-lesioned rats by comparing it with quantitative biochemical, immunohistochemical, and behavioral measurements.

**Methods:**

Different doses of 6-OHDA (0, 7, 14, and 28 μg) were unilaterally injected into the right striatum of male Sprague-Dawley rats. Dopaminergic functional activity in the striatum was assessed by [^18^F]FDOPA-PET, measurement of striatal dopamine (DA) and DA metabolite levels, tyrosine hydroxylase (TH) immunostaining, and methamphetamine-induced rotational testing.

**Results:**

Accumulation of [^18^F]FDOPA in the bilateral striatum was observed in rats pretreated with both aromatic L-amino acid decarboxylase and catechol-O-methyltransferase (COMT) inhibitors. Unilateral intrastriatal injection of 6-OHDA produced a significant site-specific reduction in [^18^F]FDOPA accumulation. The topological distribution pattern of [^18^F]FDOPA accumulation in the ipsilateral striatum agreed well with the pattern in TH-stained corresponding sections. A significant positive relationship was found between Patlak plot *K*_i _values and striatal levels of DA and its metabolites (*r *= 0.958). A significant negative correlation was found between both *K*_i _values (*r *= -0.639) and levels of DA and its metabolites (*r *= -0.719) and the number of methamphetamine-induced rotations.

**Conclusions:**

*K*_i _values determined using [^18^F]FDOPA-PET correlated significantly with the severity of dopaminergic dysfunction. [^18^F]FDOPA-PET makes it possible to perform longitudinal evaluation of dopaminergic function in 6-OHDA-lesioned rats, which is useful in the development of new drugs and therapies for Parkinson's disease (PD).

## Background

Parkinson's disease is a progressive, chronic neurodegenerative disorder with movement dysfunction that primarily affects the elderly [1]. The four main symptoms of Parkinson's disease (PD; tremor, rigidity, bradykinesia, and postural instability) grow worse over time with a severe decrease in striatal dopamine (DA) content resulting from neuron loss in the substantia nigra pars compacta (SNc) projecting into the striatum (caudate and putamen).

Treatment of PD is primarily based on DA replacement using the dopamine pro-drug, levodopa (L-DOPA). Although L-DOPA remains the most effective therapy, chronic treatment causes adverse effects, such as wearing-off phenomenon and dyskinesia [2]. Gene and stem cell therapies have attracted attention as L-DOPA alternatives. Recent studies involving rodent and nonhuman primate models of PD suggested that transplantation of dopamine neurons derived from mouse, monkey, or human embryonic stem cells and delivery of dopamine-synthesizing enzymes or glial cell line-derived neurotrophic factor genes using recombinant adeno-associated viral vector into the nigral DA neurons and/or the striatal cells can provide motor benefits[3-9].

Rats unilaterally lesioned with 6-hydroxydopamine (6-OHDA) are a useful hemi-Parkinson model for studying DA-related functions. This model also aids new drug and novel therapy research because motor deficits (e.g., drug-induced rotation) can be quantified [10]. Different PD models can be developed by varying the site of 6-OHDA injection between the medial forebrain bundle (MFB), SNc, or caudate-putamen complex (CPu). The MFB and SNc lesion models, and partial DA depletion in the CPu model, mimic advanced and global PD stages, respectively [11].

Positron emission tomography (PET) can detect impairment of dopaminergic function in living brain. The fluorinated positron-emitting analog of L-DOPA, L-3,4-dihydroxy-6-[^18^F]fluoro-phenylalanine ([^18^F]FDOPA), is one of the most widely used PET tracers for studying the brain dopaminergic system. Accumulation of [^18^F]FDOPA in the brain reflects its transport, decarboxylation, and vesicular uptake in the nigrostriatal presynaptic nerve terminals. In PD patients, [^18^F]FDOPA uptake in the striatum is reduced [12-14], and there is a negative correlation between the degree of motor deficit and [^18^F]FDOPA uptake, especially in the putamen [15-19]. Other analogs used include (+)-[^11^C]dihydrotetrabenazine ([^11^C]DTBZ), which binds specifically to vesicular monoamine transporter 2 (VMAT2) in dopaminergic synaptic vesicles, and [^18^F]β-CFT (2-β-carbomethoxy-3β-(4-fluorophenyl)tropane or WIN-35,428), a dopamine transporter (DAT)-selective radioligand. A significant correlation between [^11^C]DTBZ-binding and clinical motor asymmetry and a clear association of the severity of rigidity and hypokinesia with reduced [^18^F]β-CFT uptake in the putamen have been reported [20], underscoring the usefulness of PET for PD diagnosis and assessment of lesion severity.

Recent developments in small animal PET imaging instrumentation have enabled noninvasive, quantitative, and repetitive visualization of biological functions in living animals. From a translational research perspective, *in vivo *molecular imaging with small animal disease models bridges the gap between laboratory research and human clinical studies. Many PET studies of presynaptic dopaminergic function in 6-OHDA-lesioned rats have been reported [21-32]. Inaji et al. [28] reported a significant negative hyperbolic correlation between the number of methamphetamine-induced rotations and the binding activity of [^11^C]PE2I, a selective DAT radioligand. Though DAT ligands are reportedly superior for detecting decreases in DA neurons [33-35], [^18^F]FDOPA enables examination of dopaminergic neuron functions including transport, decarboxylation, and vesicular uptake, which is useful for assessing the neuroprotective effects of therapeutic agents, as well as evaluating the differentiation of grafted cells in living brains. Especially, in the cell transplantation experiments into the striatum or gene therapy experiments, it is considered that evaluation of overall dopaminergic neuron functions using [^18^F]FDOPA-PET is useful for interpreting these therapeutic effects on differentiation or enhancement of dopaminergic neurons, whereas the accumulation of DAT ligands in the striatum means the expression of functional DAT protein, which is nothing more than a proof of the function of DA reuptake.

The severity of lesions induced by 6-OHDA injection is generally evaluated by biodistribution analysis, *ex vivo *autoradiography, or assessment of rotational behavior [33,36]. A few rodent PD model studies using [^18^F]FDOPA-PET which present no images of the specific uptake of [^18^F]FDOPA were reported to date [23,27]. Therefore, quantitative assessment of lesion severity using [^18^F]FDOPA-PET has also not been reported. In this study, we quantitatively analyzed [^18^F]FDOPA-PET in unilaterally 6-OHDA-lesioned rats, and report the first accurate evaluation of the severity of dopaminergic dysfunction using [^18^F]FDOPA-PET.

## Methods

### Chemicals

6-OHDA (Sigma Chemical Co., St. Louis, MO, USA) was dissolved in saline (Otsuka Pharmaceutical Co., Ltd., Tokyo, Japan) containing 0.2% (*w*/*v*) L-ascorbic acid (Wako Pure Chemical Industries, Ltd., Osaka, Japan). Methamphetamine sulfate (Dainippon Sumitomo Pharma Co. Ltd., Osaka, Japan) was dissolved in saline. Entacapone (Novartis Pharma, Tokyo, Japan), a peripheral catechol-O-methyltransferase (COMT) inhibitor, and carbidopa (Sigma), a peripheral aromatic L-amino acid decarboxylase (AADC) inhibitor, were suspended in 0.25% sodiumcarboxymethylcellulose. All solutions and suspensions were prepared fresh.

### Labeling synthesis

[^18^F]FDOPA was synthesized according to a slightly modified previously reported method [37]. Briefly, 4-O-pivaloyl-L-dopa (PharmaSynth As, Tartu, Estonia) was dissolved in acetic acid and acetyl [^18^F]hypofluorite was bubbled through the solution at 130°C for 10 min. The reaction mixture was then hydrolyzed in 6 M HCl at room temperature for 3 min. The product was purified using high-performance liquid chromatography (HPLC) on a COSMOSIL 5C_18_-MS-II column (20 mm (i.d.) × 250 mm, 5-μm, Nacalai Tesque, Kyoto, Japan) using 0.1% acetic acid as the mobile phase. The purified fraction was mixed with 25% ascorbic acid (Ascorbic Acid Injection USP; I'rom Pharmaceutical Co., Ltd., Tokyo, Japan) and sterilized by filtration before administration. Each solution was prepared on the day of the experiment. The pH of the final solution for animal use was around 6.0. Specific radioactivity at the time of injection ranged from 11 to 70 MBq/μmol. The radiochemical purity was over 99.5%.

### Animals

Thirty-five 6-OHDA unilaterally lesioned male Sprague-Dawley rats were purchased from Japan SLC, Inc. (Shizuoka, Japan). The animals were maintained in a temperature- and light-controlled environment with standard food and tap water provided *ad libitum*. All experimental protocols were approved by the Ethics Committee on Animal Care and Use of the Center for Molecular Imaging Science in RIKEN, and were performed in accordance with the Principles of Laboratory Animal Care (NIH publication no. 85-23, revised 1985).

### Unilateral 6-OHDA lesioning

Stereotaxic surgery was performed at Japan SLC as follows. Eight-week-old animals were anesthetized with pentobarbital sodium (50 mg/kg, i.p.) and placed in the stereotaxic head holder so that the difference in the DV value between bregma and lambda was ≤ 0.1 mm. Two microlitres of 6-OHDA solution were injected into each of four sites in the right striatum (site 1: AP +1.3, ML -2.6, DV -5.0 mm; site 2: AP +0.4, ML -3.0, DV -5.0 mm; site 3: AP -0.4, ML -4.2, DV -5.0 mm; site 4: AP -1.3, ML -4.5, DV -5.0 mm relative to bregma and ventral from the dura) at a rate of 1 μl/min using a 10-μl Hamilton microsyringe. After injection, the needle was left in place for 1 min before being slowly withdrawn. The total doses of 6-OHDA (free base) were 7 μg (*n *= 10), 14 μg (*n *= 10), and 28 μg (*n *= 11). Sham-treated control animals (*n *= 4) were injected with saline instead of 6-OHDA but otherwise treated identically.

### PET scans

A microPET Focus220 scanner (Siemens, Knoxville, TN, USA) with a 1.4-mm spatial resolution (FWHM) at the center of a view with 220 mm diameter and an axial extent of 78 mm was used for all PET scans. Rats were selected randomly and placed into groups of six animals. The sham-operated control group was composed of three animals. At 6 to 12 weeks after 6-OHDA lesioning, rats weighing 397 to 489 g were anesthetized and maintained with a mixture of 1.5% isoflurane and nitrous oxide:oxygen (7:3). Body temperature was monitored during anesthesia and maintained at 37°C. At the start of the emission scan, a single bolus of [^18^F]FDOPA was administered via the tail vein at a dose of 108 to 151 MBq/kg, representing a calculated chemical amount of [^18^F]FDOPA of 0.74 to 5.57 μmol/animal. Thirty minutes before tracer administration, rats were premedicated with carbidopa (10 mg/kg, i.p.) or both carbidopa (10 mg/kg, i.p.) and entacapone (10 mg/kg, i.p.) [33]. A minimum 1-week washout separated PET scans in the same rat.

A 3D list-mode emission scan was performed for 90 min and sorted into 41 frame-dynamic sinograms as follows: 6 × 10 s, 6 × 30 s, 11 × 60 s, 15 × 180 s, 3 × 600 s. PET images were reconstructed using microPET manager 2.4.1.1 (Siemens, Knoxville, TN, USA) by Fourier Rebinning and standard 2D filtered back projection (FBP) using a ramp filter with a cutoff at the Nyquist frequency.

### Image analysis

Images were analyzed using PMOD software (version 3.15; PMOD Technologies Ltd., Zurich, Switzerland). MRI images were collected 7 weeks after rats were 6-OHDA unilaterally lesioned and were manually aligned in space using a rat brain atlas [38]. MRI images of control rats of the same age were coregistered with those of lesioned rats. Using the rat brain atlas for guidance, regions of interest (ROI) were placed on each striatum and the cerebellum on the aligned MRI template. Each region was primarily drawn on coronal slices and then confirmed on sagittal and horizontal slices. PET images of lesioned and control rats were manually coregistered to the corresponding MRI templates. All related ROIs were stacked into the volume of interest (VOI), and the mean value in each VOI was used to generate regional time-activity curves (TACs). TACs for each tissue were constructed by normalizing decay-corrected time-radioactivity measurements to the standardized uptake value (SUV) of [^18^F]FDOPA.

### Kinetic analysis

Uptake of [^18^F]FDOPA in normal or lesioned striatum tissues was calculated by Patlak plots [39] using PMOD software. The activity of a reference TAC devoid of tracer trapping replaced the blood activity. The radioactivity profile from 10 to 60 min after [^18^F]FDOPA administration was used. The [^18^F]FDOPA influx constant was determined using the following equation:

$$\frac{C_{\text{Tissue}}\left(t\right)}{C_{\text{Reference}}\left(t\right)}=K_{\text{i}}\frac{\overset{t}{\underset{0}{\int }}C_{\text{Reference}}\left(u\right)du}{C_{\text{Reference}}\left(t\right)}+V,$$

where *C*_Tissue_(*t*) represents the tissue radioactivity concentration at time *t *as determined by PET image analysis. *C*_Reference_(*t*) represents the reference tissue (cerebellum) radioactivity concentration at time *t *as determined by PET image analysis. *K*_i _represents the [^18^F]FDOPA influx constant calculated from the regression slope. *V *represents the initial distribution volume in the tissue of interest at time 0, calculated from the Patlak plot *y*-intercept.

### HPLC analysis of radio-metabolites in plasma

Plasma radio-metabolites were analyzed to confirm the effect of pretreatment with carbidopa and entacapone on the metabolism of [^18^F]FDOPA by peripheral COMT and DDC. Venous blood was collected from the tail vein after the emission scan and centrifuged at 12 000 rpm for 2 min at 4°C. A fivefold volume of cold 0.2 M perchloric acid containing 100 μM Na_2_EDTA was then added and the resulting suspension was centrifuged at 15,000 rpm for 15 min at 4°C, filtered through a 0.45 μm PTFE filter (Millipore, Billerica, MA, USA), and an aliquot was injected onto a HPLC system (Shimadzu Corporation, Kyoto, Japan) with a UG-SCA30 NaI(TI) positron detector (Universal Giken, Kanagawa, Japan). Radio-metabolites were separated on an EICOMPAK SC-5ODS column (3.0 mm (i.d.) × 150 mm; Eicom Corporation, Kyoto, Japan) using 0.1 M sodium citrate-acetate buffer (pH 3.5) containing 17% methanol, 0.88 mM sodiumoctanesulfonic acid, and 0.013 mM Na_2_EDTA as the mobile phase. The analysis was carried out at a column temperature of 25°C and a flow rate of 0.5 ml/min. Major ^18^F peaks were identified by comparing retention times with those of authentic metabolite standards. The individual peak areas of the radiometric chromatograms were corrected for radioactive decay to the retention time of the radio-metabolite for each sample and expressed as fractions of total radioactivity.

### Monoamine quantitation

At least 6 days separated PET scanning and sacrificing for the determination of monoamine level in the striatum, in the meantime we performed the post-scan processes including reconstruction, image analysis, kinetic analysis, and metabolite analysis to confirm the successes of PET scanning. In addition, behavioral abnormality and level of tyrosine hydroxylase (TH) immunohistochemistry were stable from 6 to 18 weeks after the unilaterally 6-OHDA-lesioned rats (our personal data), indicating that a period between PET scanning and sacrificing, within 6 days, may not be critically influenced on the results using the animals within 18 weeks after the lesion. At 10 to 16 weeks after lesioning, animals (control group: *n *= 4; 7 μg group: *n *= 9; 14 μg group: *n *= 9; 28 μg group: *n *= 7) were anesthetized with 1.5% isoflurane and decapitated. Each brain was rapidly removed, rinsed in chilled saline, and dissected from the lesioned and unlesioned striata over ice. Striatal tissues were transferred into pre-weighed plastic tubes and stored at -80°C until use.

To measure the levels of monoamines and the metabolites DA, 3,4-dihydroxyphenylacetic acid (DOPAC), homovanilic acid (HVA), serotonin (5-HT), and 5-hydroxyindoleacetic acid, striatal tissue was homogenized for 30 s in 0.2 M perchloric acid containing 100 μM Na_2_EDTA, kept on ice for at least 30 min, then centrifuged at 20,000 × *g *at 4°C for 15 min. The supernatant was diluted twofold in 0.2 M perchloric acid containing 100 μM Na_2_EDTA, 30 ng of isoproterenol internal standard was added, and the pH was adjusted to approximately 3.0 with 1 M sodium acetate. The resulting solution was filtered (0.45 μm PTFE filter, Millipore) and 10 μl was analyzed by HPLC. DA, 5-HT, and their metabolites were electrochemically detected using an amperometric detector (ECD-700S; Eicom Corporation, Japan) by oxidation on a graphite electrode at 0.75 V relative to the Ag-AgCl reference electrode. The amount of monoamines (expressed as nanomoles per gram tissue) was determined by comparing the ratio of the monoamine peak area to that of the internal standard applied to calibration curves.

### Tyrosine hydroxylase immunohistochemistry

One representative rat from each 6-OHDA-lesioned group except the control was used for TH immunohistochemistry at 12 to 13 weeks after lesioning. Rats were deeply anesthetized with pentobarbital and perfused transcardially with saline containing heparin (10 units/ml) followed by 4% paraformaldehyde in 0.1 M phosphate buffer (pH 7.4). Brains were removed and fixed in the same fixative at 4°C for 2 days. Tissues were cryoprotected in 15% to 30% sucrose solution and frozen on dry-ice. Sections of 40 μm were cut using a sliding microtome (Retoratome REM-700, Yamato Kohki Industrial Co., Ltd., Saitama, Japan) with a refrigeration unit (Electro Freeze MC-802A, Yamato Kohki Industrial Co., Ltd.). Sections were heated in antigen retrieval solution (0.01 M citrate buffer, pH 6.0) at 90°C for 15 min, chilled in water, then immersed for 5 min in PBS containing 0.3% Triton X-100 (TPBS). Endogenous peroxidase activity was quenched for 30 min in PBS containing 0.3% H_2_O_2_. After three 5-min washes in TPBS, sections were preincubated with blocking solution (Block Ace™; DS Pharma Biomedical Co., Ltd., Osaka, Japan) at room temperature for 60 min and incubated overnight at 4°C with a 1:30,000 dilution of mouse anti-TH monoclonal antibody (ImmunoStar, Inc., Hudson, WI, USA) in blocking solution. Sections were washed with TPBS for 5-min three times and incubated with peroxidase-labeled anti-mouse IgG-specific secondary antibody (Nichirei Histofine^® ^Simple Stain Rat MAX PO (M), Nichirei Biosciences Inc., Tokyo, Japan) at room temperature for 120 min. After three 5-min washes with TPBS, the reaction was visualized using 3,3-diaminobenzidine as a chromogen. Dehydrated sections were mounted on glass slides and covered with coverslips.

### Rotational behavior

All rats were tested for rotational behavior induced by methamphetamine (2.5 mg/kg, i.p.) at 8 to 9 weeks after lesioning. Rotational behaviors were recorded for 90 min with a video camera. Full 360° turns in the direction ipsilateral to the lesion (clockwise) were counted. A minimum 4-day washout period separated PET scanning and behavioral testing.

### Statistical analysis

All results were expressed as the mean ± S.D. Conventional statistical analyses were carried out using GraphPad Prism (version 5.02, GraphPad Software, Inc., San Diego, CA, USA). The paired Student's *t *test was used to compare *K*_i _values and the levels of tissue DA and its metabolites in the lesioned and contralateral sides within the same dose groups. Inter-group comparisons involved one-factor analysis of variance (ANOVA) followed by post hoc Dunnett's testing. Statistical significance was set at *P *< 0.05. Correlation between measurements was analyzed by linear regression using data from rats in which values for all three measures were available.

## Results

### Effect of carbidopa and entacapone on striatal accumulation of [^18^F]FDOPA

**Figure 1:**
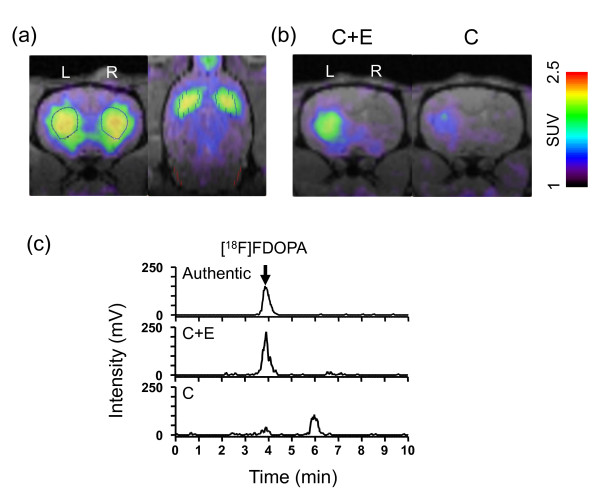
**[^18^F]FDOPA-PET images of rats and radiochromatograms of rat plasma samples after the administration of [^18^F]FDOPA**. **(a) **Representative [^18^F]FDOPA-PET image of a sham-operated control rat coregistered with MRI template at 7 weeks after sham operation. PET image was reconstructed using FBP and summated from 11 to 90 min after the tracer injection. Blue broken lines in both panels and red lines in right panel indicate ROIs (striatum and cerebellum, respectively). L = left striatum; R = right striatum. **(b) **Effect of carbidopa (C) and entacapone (E) on striatal accumulation of [^18^F]FDOPA in 6-OHDA-lesioned rats. PET images were summated from 11 to 90 min after tracer injection. One rat from the 6-OHDA 28 μg group was scanned in the presence of both inhibitors (left panel, "C + E"), and after 2 weeks the same rat was scanned in the presence of carbidopa (right panel, "C"). L = left striatum; R = right striatum. **(c) **Radiochromatograms of authentic [^18^F]FDOPA and plasma obtained from each rat in (b) after PET scan completion. [^18^F]FDOPA (peak at 3.8 min) was detected in the plasma from the rat pretreated with carbidopa and entacapone (C + E), whereas it was low and the other metabolite, may be 3-O-methyl-FDOPA, existed mainly (peak at 6.0 min) in the plasma from the rat pretreated with carbidopa only (C).

[^18^F]FDOPA-PET studies using sham-operated, unlesioned control rats pretreated with carbidopa and entacapone indicated that [^18^F]FDOPA accumulates highly in the bilateral striatum rather than other regions, including the cerebellum. In rats unilaterally lesioned with 28 μg of 6-OHDA, [^18^F]FDOPA uptake in the striatum was significantly lower in the ipsilateral (right) side compared to the contralateral (left) side of the injection (Figure 1b, "C + E"). The difference was larger in rats pretreated with carbidopa and entacapone than in rats pretreated with carbidopa only (Figure 1b, "C"). Figure 1c shows the radiochromatograms of [^18^F]FDOPA in the plasma of the rat shown in Figure 1b collected after PET scanning. One major peak with a retention time identical to that of authentic [^18^F]FDOPA (3.8 min) was detected in the plasma from the rat pretreated with carbidopa and entacapone. This peak was small in the plasma from the rat pretreated with carbidopa only, and there was a later peak with a retention time identical to that of authentic 3-O-methyl-FDOPA (OMFD) (6.0 min) (data not shown).

### Correlation between PET measurements of [^18^F]FDOPA uptake and TH immunohistochemistry

**Figure 2:**
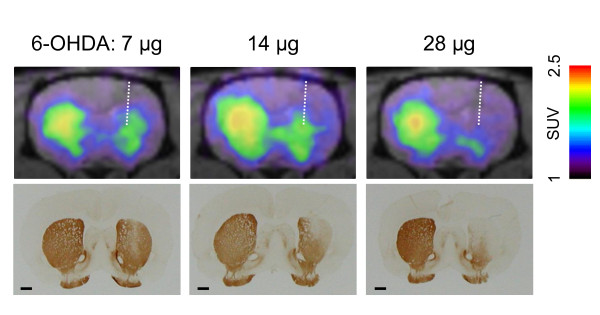
**Comparison between [^18^F]FDOPA-PET images and TH immunostaining**. Upper panels show summated coronal PET images (from 11 to 90 min) of representative rats injected with 7 μg (left), 14 μg (middle), and 28 μg (right) of 6-OHDA. Lower panels show photomicrographs of TH-immunostained sections corresponding to PET images from the same rats. Scale bar = 100 μm.

Figure 2 shows coronal PET images and corresponding photomicrographs of TH-immunostained sections from 6-OHDA-injected rats. Uptake of [^18^F]FDOPA in the contralateral (left) striatum was high and similar in all rats, while uptake was low in the ipsilateral (right) striatum and along the 6-OHDA injection site, especially in the dorsal part of the striatum, which was consistent with immunostaining results.

### PET assessment of 6-OHDA-induced dopaminergic denervation

**Figure 3:**
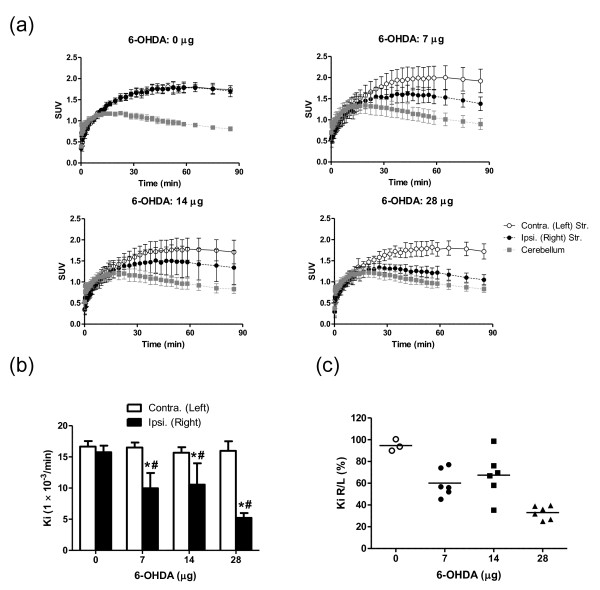
**Effect of 6-OHDA on time profiles of the tissue radioactivity in brain and *K*_i _values**. After the administration of [^18^F]FDOPA in rats. **(a) **Time-activity curves for the radioactivity in the contralateral striatum, ipsilateral striatum, and cerebellum. **(b) ***K*_i _values in the bilateral striata of the different dosage groups (0 μg, *n *= 3; 7, 14, and 28 μg, *n *= 6). Asterisk (*): significant difference vs. the contralateral side; paired Student's *t *test; number sign (#): significant difference vs. the 0 μg group, one-way ANOVA followed by Dunnett's multiple comparison test. **(c) ***K*_i _values expressed as the percentage ratio of the ipsilateral (right) to the contralateral (left) side for each 6-OHDA dose group.

Time-activity curves indicated decreased accumulation in the ipsilateral (right) striatum compared to the contralateral (left) striatum, suggesting that 6-OHDA administration induced degeneration of the dopaminergic synaptic terminals (Figure 3a). Values of *K*_i_, the [^18^F]FDOPA influx constant, were significantly lower for the ipsilateral striatum compared to the contralateral side. One-way ANOVA revealed that 6-OHDA treatment significantly reduced the *K*_i _values for the ipsilateral striatum (*F *= 14.27, *P *< 0.0001), but not for the contralateral striatum (Figure 3b). The individual *K*_i _right/left ratios (*K*_i _R/L) were plotted against the 6-OHDA dose (Figure 3c). The mean *K*_i _R/L of the 6-OHDA groups were lower than the control group, but in the 7- and 14-μg-dose groups, individual values were scattered, indicating that the degree of denervation varied among these rats.

### Biochemical assessment of 6-OHDA-induced dopaminergic denervation

**Figure 4:**
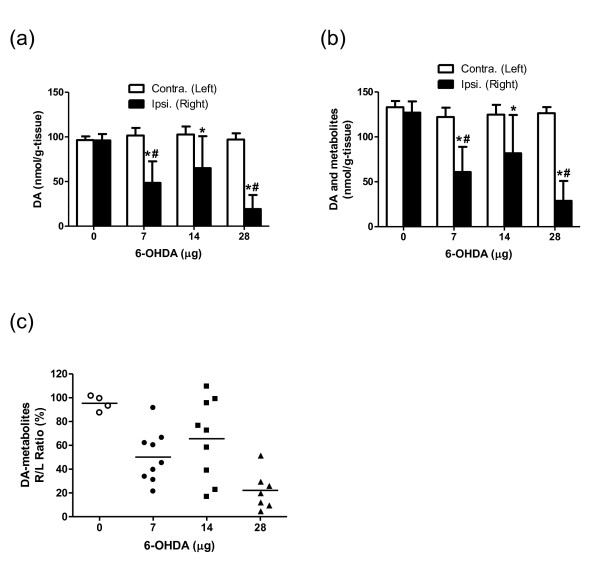
**Dose profiles of 6-OHDA on the striatal DA and its metabolite contents**. The influence of 6-OHDA on the striatal level of **(a) **DA, **(b) **DA and its metabolites, and **(c) **DA and its metabolites in each animal expressed as the percentage ratio of the ipsilateral (right) to the contralateral (left) side. 6-OHDA: 0 μg, *n *= 4; 7 and 14 μg, *n *= 9; 28 μg, *n *= 7. Asterisk (*): significant difference vs. the contralateral side, paired Student's *t *test; number sign (#): significant difference vs. 0 μg group, one-way ANOVA followed by Dunnett's multiple comparison test.

The DA content in the ipsilateral striatum was significantly lower than in the contralateral striatum in the 6-OHDA-injected groups (Figure 4a). Inter-group comparison by ANOVA revealed that DA levels on the ipsilateral side were significantly reduced by 6-OHDA treatment. Dunentt's posthoc comparisons indicated a significant decrease in DA levels in the 7- and 28-μg groups compared to the control group, but no difference in the contralateral striatum. As shown in Figure 4b, a similar tendency was observed when the levels of DA and DA metabolites were summed, indicating that DA was the major catecholamine component of the striatum. Three kinds of DA-metabolites (DOPAC, HVA, and 3-O-methyltyramine (3-MT)) were detected in the striatum. DOPAC (10% to 24% of catecholamine component) was more abundant than HVA and 3-MT in the striatum (3.6% to 6.4% and 2.7% to 4.0%, respectively). The percentage ratio of the level of DA and DA-metabolites in the right and left striatum (DA-metabolites R/L) plotted against the 6-OHDA dose was lower in the 6-OHDA groups compared to the control group (Figure 4c). In the 7- and 14-μg-dose groups, interindividual differences were large (Figure 3c), indicating a highly variable degree of striatal dopaminergic denervation in these animals.

### Correlation between PET, biochemical, and behavioral measurements

**Figure 5:**
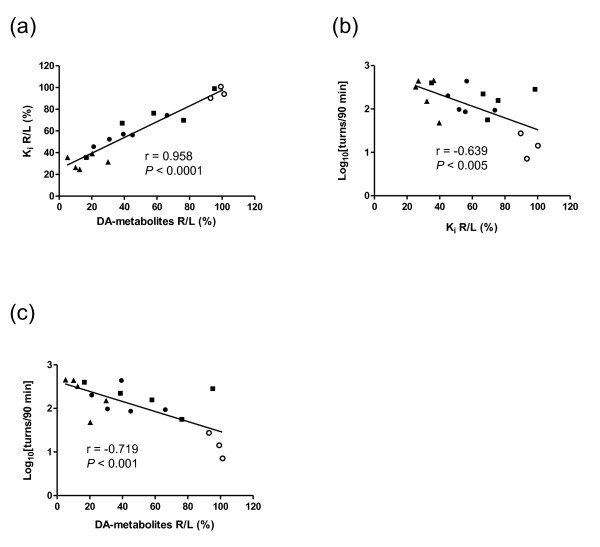
**Correlation among [^18^F]FDOPA-PET, biochemical measurements, and behavioral index**. **(a) **The striatal levels of DA and its metabolites (% of the contralateral side value) vs. the *K*_i _(% of the contralateral side value) in the PET study. 6-OHDA: 0 μg, *n *= 3; 7, 14, 28 μg, *n *= 5. **(b) ***K*_i _(% of the contralateral side value) vs. the logarithm of the number of methamphetamine-induced rotations. 6-OHDA: 0 μg, *n *= 3; 7, 14, 28 μg, *n *= 5. **(c) **The striatal levels of DA and its metabolites (% of the contralateral side value) vs. the logarithm of the number of methamphetamine-induced rotations. 6-OHDA: 0 μg, *n *= 3; 7, 14, 28 μg, *n *= 5. Symbols show the values of 0 μg (open circle), 7 μg (closed circle), 14 μg (closed square), and 28 μg (closed triangle) for each individual.

Figure 5a illustrates the significant correlation between the *K*_i _R/L and the DA-metabolites R/L. The *K*_i _R/L negatively correlated with the common logarithm of the number of methamphetamine-induced rotations (Figure 5b), and the DA-metabolites R/L negatively correlated with the behavioral measurements (Figure 5c).

## Discussion

Ours is the first report of the use of [^18^F]FDOPA-PET imaging in a rat PD model to discriminate the striatum from other cerebral structures. Although [^18^F]FDOPA is widely used in primate PET studies, there are few reports of its use in rodents and there are no specific PET images of [^18^F]FDOPA accumulation in the striatum in these reports [23,27].

[^18^F]FDOPA is primarily metabolized to [^18^F]fluorodopamine ([^18^F]FDA) by AADC in the peripheral tissues and to [^18^F]OMFD by COMT [40]. Therefore, in order to ameliorate the accumulation of radiotracer in the striatum, co-administration of the AADC inhibitor carbidopa and peripheral COMT inhibitor entacapone was necessary prior to [^18^F]FDOPA-PET (Figure 1) [33]. In addition, the high spatial resolution of micro PET and superior image reconstruction process we used enabled successful PET imaging with [^18^F]FDOPA in rodents [41].

Radio-metabolite analysis of plasma sampled after the emission scan (90 min) revealed that [^18^F]FDOPA was almost intact in rats pretreated with carbidopa and entacapone, but was metabolized to [^18^F]OMFD in rats treated only with carbidopa (Figure 1). Pauwels et al. [42] showed that [^18^F]FDA (the major metabolite), [^18^F]FDOPA, [^18^F]OMFD, and [^18^F]FDOPAC are present in the striatum of rats pretreated with both carbidopa and entacapone, while [^18^F]OMFD is the major striatum metabolite in rats pretreated only with carbidopa 30 min after [^18^F]FDOPA injection. Therefore, the same components of ^18^F-radioactivity might have contributed to the striatum radioactivity we observed.

The topological distribution of [^18^F]FDOPA accumulation we observed in both the dorsal and ventral striatum (nucleus accumbens) agreed well with TH immunostaining of the corresponding sections, indicating that small animal PET imaging can provide detailed information regarding tracer location in the brain. This is particularly useful for evaluating the dopaminergic function of grafted cells because the accumulation of [^18^F]FDOPA at graft sites would suggest the existence of TH-positive neurons that have differentiated from grafted cells.

Values of the [^18^F]FDOPA influx rate constant *K*_i _for the left striatum of all groups and the right striatum of the control group ranged from 15.7 to 16.7 × 10^-3 ^min^-1^. Using a noninvasive approach to determine *K*_i_, Takikawa *et al. *reported a mean *K*_i_^OCC ^value of 6.6 ± 1.8 × 10^-3 ^min^-1 ^in healthy volunteers pretreated with carbidopa [19]. In baboons without pretreatment, the mean *K*_i _values for the caudate and putamen of control animals were 6.9 ± 1.2 and 7.7 ± 1.8 × 10^-3 ^min^-1 ^[35]. The *K*_i _value also reflects the blood-brain barrier (BBB) influx rate and the transport function of system L, which is composed of the L-type amino acid transporter 1 (LAT1) and 4F2hc [43]. System L transports large neutral amino acids including leucine, tryptophan, tyrosine, phenylalanine, and amino acid related drugs such as L-DOPA, alpha-methyldopa, melphalan, and gabapentin [44]. Our results suggest that L-DOPA transport across the rat BBB is greater than transport across the human BBB, which is consistent with a previous study showing that the mean rate of phenylalanine uptake and levels of LAT1 and 4F2hc in isolated brain microvessels are lower in humans than in rodents [45].

It is noteworthy that there was a significant positive correlation between the *K*_i _R/L and the striatal DA-metabolites R/L, although with both of these parameters there was no clear dose-dependency in the 7- and 14-μg 6-OHDA groups. Postmortem measurements revealed that the levels of DA and its metabolites in the striatal lesions varied between the 7- and 14-μg groups. Our data thus suggest that [^18^F]FDOPA-PET is also useful for screening striatal lesions (e.g., prior to cell transplantation experiments).

Although the right/left ratio of *K*_i _values correlated well with striatal levels of DA and its metabolites, the linear regression curve did not pass through the origin. Even in the 28 μg group, which showed the most severe lesions, the right/left ratio of *K*_i _values ranged from 25.0% to 39.5%, whereas the striatal DA-metabolites R/L ranged from 4.9% to 29.8%. Similarly, Ito *et al. *[34] reported that the regression intercept in plots of the remaining percentage of DA neurons in SNc versus percent of striatal [^14^C]L-DOPA-specific uptake did not intersect with the origin. One possible explanation for this result is that the [^18^F]FDOPA-PET *K*_i _value reflects its transport and decarboxylation not only by the dopaminergic neurons, but also by serotonergic terminals, capillary endothelium, pericytes, and different types of non-monoaminergic neurons in the striatum, since these have been reported to show DDC activity and to be sites of L-DOPA conversion to DA after dopaminergic denervation [46-56].

As shown Figure 5b, the *K*_i _R/L values correlated linearly with the common logarithm of the number of rotations induced by methamphetamine. A similar correlation between percent of striatal [^14^C] L-DOPA-specific uptake and the number of methamphetamine-induced rotations was reported by Ito and colleagues [34]. We also showed that the striatal DA-metabolites R/L values correlated with the behavioral measurements, which is consistent with previous reports [57,58]. These results indicate that [^18^F]FDOPA-PET is ideal for noninvasive, accurate quantitative evaluation of dopaminergic function. It might be quite valuable to measure the recovery of dopaminergic function in cell transplantation experiments using 6-OHDA-lesioned rats, since discrepancies between recovery of motor deficit and the number of TH^+ ^cells counted in postmortem analysis have been reported [59,60]. Considering such animal-to-animal variation, longitudinal *in vivo *PET imaging in individual grafted rats would be quite informative.

## Conclusions

This is the first report demonstrating the utility of [^18^F]FDOPA-PET for imaging dopaminergic function in unilaterally 6-OHDA-lesioned rats. The severity of the dopaminergic denervation determined by [^18^F]FDOPA-PET correlated well with that determined by widely used biochemical, immunohistological, and behavioral assays. [^18^F]FDOPA-PET enables noninvasive, quantitative evaluation of dopaminergic function in 6-OHDA-lesioned rats and will be a useful tool in the development of new therapies for PD.

## Competing interests

The authors declare that they have no competing interests.

## Authors' contributions

All authors accept full responsibility for the accuracy of the raw data and data analysis, and exercised authority in manuscript preparation and the decision to submit the manuscript for publication. KK designed and performed all animal experiments, analyzed the data, and wrote the manuscript. TT helped the study design, animal experiments, and kinetic analysis and critically reviewed the manuscript. YKat helped animal experiments, radio-metabolite analysis, and monoamine quantitation. TK assisted in conducting rotational behavior test and gave valuable input on biological aspects. RZ and KT contributed to the labeling synthesis of [^18^F]FDOPA. MG performed tyrosine hydroxylase immunohistochemical study. YKuw provided intellectual input, performed data quality assurance of all the experiments. YWad helped perform PET scan and image reconstruction. HO is the co-PI of this study and involved in the design and interpretation of the analysis as well as in writing manuscript. YWat is the PI of the study and is involved in all aspects of this work from design to writing and approving the final content of the manuscript.
